# Draft Genome Sequences of Two Genetic Variant Strains of *Edwardsiella piscicida*, JF1305 and RSB1309, Isolated from Olive Flounder (*Paralichythys olivaceus*) and Red Sea Bream (*Pagrus major*) Cultured in Japan, Respectively

**DOI:** 10.1128/genomeA.00546-14

**Published:** 2014-06-12

**Authors:** Kazuki Oguro, Kazuki Tamura, Jin Yamane, Masato Shimizu, Takeshi Yamamoto, Takuya Ikawa, Kouhei Ohnishi, Syun-ichirou Oshima, Masayuki Imajoh

**Affiliations:** a Laboratory of Fish Disease, Faculty of Agriculture, Kochi University, Nankoku, Kochi, Japan; b Azuma-cho Fisheries Cooperative Association, Nagashima, Izumi, Kagoshima, Japan; c Graduate School of Kuroshio Science, Kochi University, Nankoku, Kochi, Japan; d Research Institute of Molecular Genetics, Kochi University, Nankoku, Kochi, Japan

## Abstract

*Edwardsiella piscicida* is a new species discovered within the group of organisms traditionally classified as *Edwardsiella tarda*. We present draft genome sequences of two variant strains of *E. piscicida*, JF1305 and RSB1309. Differences in protein-coding sequence between these isolates are associated with virulence, disease, and defense, suggesting differences in pathogenicity.

## GENOME ANNOUNCEMENT

*Edwardsiella tarda* is a Gram-negative, rod-shaped, facultative anaerobic bacterium belonging to the family *Enterobacteriaceae*. In Japan, this bacterium causes edwardsiellosis in economically important cultured fish species, including olive flounder (*Paralichthys olivaceus*) (1) and red sea bream (*Pagrus major*) (2). There are as yet no effective measures to control the disease (3). *E. tarda* isolates from red sea bream are designated as atypical *E. tarda* because the phenotypic characteristics differ from those of typical *E. tarda* from freshwater fishes and olive flounder (2). Moreover, there are differences in pathogenicity between atypical and typical *E. tarda* strains (4).

A new species designated as *Edwardsiella piscicida* was recently discovered within the group of organisms traditionally classified as *E. tarda* (5). This species includes two genetic variants, *E. piscicida* and *E. piscicida*-like species. Griffin et al. (6) reported that 44 isolates identified previously as *E. tarda* in the United States belong to *E. piscicida*. They also suggested that what Sakai et al. (7) referred to as typical and atypical *E. tarda* and what Abayneh et al. (5) described as *E. piscicida* and *E. piscicida*-like species are synonymous, which suggests that *E. piscicida* may be a potential pathogen for fish species cultured in Japan.

We isolated *Edwardsiella* in May 2013 from olive flounder cultured in Kagoshima Prefecture, Japan, and in September 2013 from red sea bream cultured in Kochi Prefecture, Japan. The species of the two *Edwardsiella* isolates were examined by PCR assay with published species-specific primers (6). Based on the PCR results, the isolates from olive flounder and red sea bream were classified as *E. piscicida* and *E. piscicida*-like species, respectively. The two genetic variant strains of *E. piscicida*, JF1305 and RSB1309, were cultured in 10 ml brain heart infusion broth overnight at 27°C. The genomic DNA was extracted with a Qiagen Genomic-tip 500/G kit and a genomic DNA buffer set. Genome sequencing was performed on a 454 GS Junior System (Roche), which generated 132,085 reads for strain JF1305 and 162,723 reads for strain RSB1309. The sequencing reads were assembled with GS De Novo Assembler version 2.9 software (Roche). The assembly of strain JF1305 consisted of 72 contigs (>500 bp) totaling 3,716,823 bp, with a G+C content of 59.8%. The assembly of strain RSB1309 consisted of 38 contigs (>500 bp) totaling 3,838,654 bp, with a G+C content of 59.3%. These draft genome sequences were annotated using the Microbial Genome Annotation Pipeline (http://www.migap.org/), yielding 3,351 protein-coding sequences (CDSs), 73 tRNA genes, and 4 rRNA operons for strain JF1305 and 3,480 CDSs, 79 tRNA genes, and 4 rRNA operons for strain RSB1309. Furthermore, the Rapid Annotations using Subsystems Technology server (8) was used. There were 73 CDSs for strain JF1305 and 65 CDSs for strain RSB1309 involved in virulence, disease, and defense, respectively. These findings suggested differences in pathogenicity between the two genetic variants of *E. piscicida*, similar to those reported between typical and atypical *E. tarda* strains (4). Further studies on putative pathogen-associated proteins are needed to understand the mechanisms underlying the pathogenicity of *E. piscicida*.

### Nucleotide sequence accession numbers.

These whole-genome shotgun projects have been deposited in GenBank under the accession no. BAYT00000000 for strain JF1305 and BAYU00000000 for strain RSB1309. The versions described in this paper are the first versions, BAYT01000000 and BAYU01000000, respectively.
